# Preliminary toxicokinetic study of BPA in lactating dairy sheep after repeated dietary and subcutaneous administration

**DOI:** 10.1038/s41598-020-63286-z

**Published:** 2020-04-16

**Authors:** Sabina Šturm, Iztok Grabnar, Andrej Škibin, Milan Pogačnik, Vesna Cerkvenik-Flajs

**Affiliations:** 1 University of Ljubljana, Veterinary Faculty, Institute of Pathology, Wild Animals, Fish and Bees, Ljubljana, 1000 Slovenia; 2 University of Ljubljana, Faculty of Pharmacy, Department of Biopharmaceutics and Pharmacokinetics, Ljubljana, 1000 Slovenia; 3 University of Ljubljana, Veterinary Faculty, Clinic of Reproduction and Farm Animals, Infrastructure Centre for Sustainable Recultivation Vremščica, Ljubljana, 1000 Slovenia

**Keywords:** Computational models, Environmental chemistry, Endocrine system and metabolic diseases

## Abstract

Dietary intake is the predominant route of human exposure to bisphenol A and one of the important food commodities is milk. The aim of our study was to preliminarily evaluate the bisphenol A exposure and disposition in sheep milk after repeated dietary and subcutaneous administration of a relatively low dose (100 µg/kg of b. w./day) of bisphenol A to a sheep. On the basis of blood plasma sampling, milk sampling and HPLC analysis, we developed the toxicokinetic model. With the toxicokinetic model we showed that most likely only free bisphenol A passes into the mammary gland and is subsequently conjugated there. The percentage of the dose eliminated with milk was less than 0.1%, regardless of the route of bisphenol A administration. It is proven that the bisphenol A is eliminated through the milk of lactating sheep. However, the amounts excreted in the milk that were detected in this study are minimal.

## Introduction

Since the start of the commercial production of bisphenol A (BPA) in the 1950s until the present, the global production and consumption of this substance, regardless of the suspected negative health effects, has continued to rise^1^. With both the wide use of BPA and its leaching from many products and materials^2^, it is known to be one of the ubiquitous environmental contaminants^3^. The main route of human BPA exposure is thought to be oral ingestion (up to 83% of the total estimated exposure), and in 2013 canned products accounted for about 50% of the dietary exposure to BPA. Thus, cans and packaging are believed to be the main source of contamination in foods^4^. Current migration limit of BPA from varnishes or coatings applied to materials and articles is 0.05 mg/kg BPA of food^5^. However, the products from farm animals, being directly exposed to human pollution, could still be, in some cases, an additional risk factor for human exposure^6^.

It is believed that BPA causes endocrine disrupting effects by the interaction with various receptors, such as thyroid hormone receptor, androgen receptor and oestrogen receptor. Thus, BPA health hazards for reproductive system, nervous system, metabolic function, immune function, the growth and development of offspring were raised^4,7^. The European Food Safety Authority (EFSA) decreased the tolerable daily intake (TDI) from the 50 μg/kg of b. w./day to 4 μg/kg of b. w./day as a response to a refined risk assessment of BPA^7^.

A food commodity important in our daily diet is milk. The quality and safety of milk depends considerably on the environment and human activity in its production. A broad range of environmental contaminants can enter the milk chain in the beginning via application of contaminated material on the soil such as industrial waste and sewage sludge, and atmospheric deposition from industrial activities^6^. It is also true that chemicals can enter milk during the collection and preparation processes of dairy products^8^. For instance, BPA may be introduced during milking from plastic parts of the milking machines, or also transferred from bulk milk to plastic storage tanks^9^. Finally, BPA can migrate as an additive from packaging material into the consumable milk. The actual levels of BPA found in commercial milk samples are presented in the review of Mercogliano and Santonicola, and are in the range between not detected (ND) to 521 ppb^6^.

To the best of our knowledge, only a few *in vivo* studies are published regarding BPA transfer to milk, with all of them using rodent models, and all report limited excretion of BPA into milk^10–13^. Doerge *et al*. evaluated the lactational transfer of BPA after repeated oral dosing in rats, and found concentrations of 0.83 +/− 0.26 nM of free BPA and 7.6 +/− 2.8 nM of total BPA 1 hour after the administration of 100 µg/kg b. w.. They calculated that doses delivered to pups lactationally were 300-fold lower than the dose administered to the dams^13^.

Cows are mainly used in milk production in Europe, accounting for 96.9% of the total milk produced^14^. However, a significant part of the agrarian economies and sheep dairy products have gained market size due to the product’s quality, high yield, and high nutritional value^15^. Sheep are also frequently used as a model for cattle and other large mammals, due to their easier manipulation. The comparable digestive physiology (polygastric model) in sheep and cows enables the assumption that the sheep model is in terms of toxicokinetics (TK) a relevant model for cows as well^16^.

The aim of our study was to estimate the transfer of BPA from feed or via subcutaneous administration to milk. To do so, one Slovenian autochthonous dairy sheep, an Istrian Pramenka, and her lamb were used in the study. Time courses of the free BPA, bisphenol A glucuronide (BPA-GLUC) and total BPA concentrations were followed in the ewe’s blood plasma after repeated dietary and subcutaneous administration, as well as BPA transfer in milk. We also aimed to assess lactational transfer of BPA to the suckling lamb by estimating BPA exposure in its blood plasma. As gastrointestinal tract of our ruminant model is very specific and the detection of BPA or BPA-GLUC in the milk was never performed after *in vivo* experiment in this species, we were unable to predict the outcomes of the study. Thus, only one animal and only one concentration gradient (100 µg/kg b. w.) was used in this preliminary study.

## Materials and methods

### Chemicals

Bisphenol A ≥ 99% purity (Merck, Sigma-Aldrich, Darmstadt, Germany) was dissolved in absolute ethanol and corn oil for the dietary (po) and subcutaneous (sc) routes of administration, respectively. The volume administered to the sheep was adjusted to the body weight recorded on the day of the administration. For the dietary administration, approximately 1 mL of BPA solution in absolute ethanol was applied onto the pellet ration to obtain the single dose of 100 µg/kg b. w., and applied with the morning feed of pellets (400 g). For the sc administration, the injection of BPA solution was performed in the shoulder area (2.9 mL) at the same dose. Both solutions were stored at the ambient temperature in sealed amber glass bottles for the entire duration of use. All materials used for the solution preparation, sample processing and assays were either made of glass or of BPA-free plastics.

The dose of 100 μg/kg b. w. was chosen in this study. As it is within the linear pharmacokinetic range at a level as close as possible to the range of proposed human exposure, yet high enough to measure both aglycone (i.e., active) and conjugated (i.e., inactive) forms of BPA in samples analyzed^13^.

### Animal husbandry

All animal procedures were carried out in accordance with ethical standards and approved by the Administration of the Republic of Slovenia for Food Safety, Veterinary Sector and Plant Protection with permission no. U34401-3/2015/8. The study was performed on one stabled, healthy lactating Istrian Pramenka sheep with a single female suckling lamb in a sheepfold at the Infrastructure Centre for Sustainable Recultivation at Vremščica belonging to the Veterinary Faculty of the University of Ljubljana, Slovenia. The ewe was six years old and weighed 59 kg, while the suckling lamb was four weeks old and weighed 12 kg. The ewe and lamb were kept under natural temperature and photoperiodic conditions, with free access to water, hay and salt. In addition, the sheep was fed twice a day with 400 g plant based pellets (SchafKorn Lac, Unser Lagerhaus Warenhandels Ges., Austria). Eventual contamination of the experimental environment was checked by preliminary testing of drinking water and pellets by high-performance liquid chromatography (HPLC) analysis, which revealed the slight presence of BPA of 0.02 µg/L and 5 µg/kg in these two matrices, respectively. The sheep and its lamb were, at both periods of the study, penned individually the day before the first administration until three days after the last administration. The lamb was kept with its mother, except on sampling days, when they were separated for a few hours before sampling time to collect enough milk for analysis. The animals were clinically healthy, as indicated by medical (temperature, breathing and rumination frequency, pulse rate) (see Supplementary Table S1), haematological and biochemical (see Supplementary Tables S2 and S3) examinations. Fourteen days after the second experimental period, the sheep and its lamb were released in their original herd.

### Experimental design

The experiment was divided into two periods, the first being the dietary administration period and the second being the subcutaneous administration period. The same ewe was used for both exposure routes. Before the start of the second period, a 13 days wash-out period was permitted to ensure that BPA was removed from the body of the ewe. In the first period, the ewe received BPA in its diet (100 µg/day/kg b. w.) for five consecutive days (dietary route of administration). The ewe ingested all pellets within 2–9 minutes. During the second period, the same ewe was injected in the shoulder area with 100 µg/kg b. w. of BPA subcutaneously per day for five consecutive days (subcutaneous route of administration).

On the first day of the dietary period of the experiment, the ewe’s blood samples were taken at time 0 (before the first administration) and at 0.083, 0.16, 0.33, 0.5, 1, 2, 4, 6, 8, 10 and 24 hours after the first administration. The blood samples were then taken every day for the next seven days (trough concentrations). The sampling time started when the ewe ingested the whole portion of pellets. Similar sampling intervals were used in the second subcutaneous period of the experiment, with the exception of the first blood sampling (0.083 h after the sc administration), which was not taken. Blood samples from the suckling lamb were collected on the first day 10 hours after BPA administration to the ewe, and on every following day before the next administration to the ewe. Jugular vein blood samples were collected in heparinised glass vacuum tubes, cooled to 4 °C and transported to the laboratory where blood plasma was separated by centrifugation at 2640 × g for 15 min. The plasma was transferred and stored in polypropylene (PP) tubes. Plasma samples were kept frozen at −20 °C until analysis.

**Figure 1:**
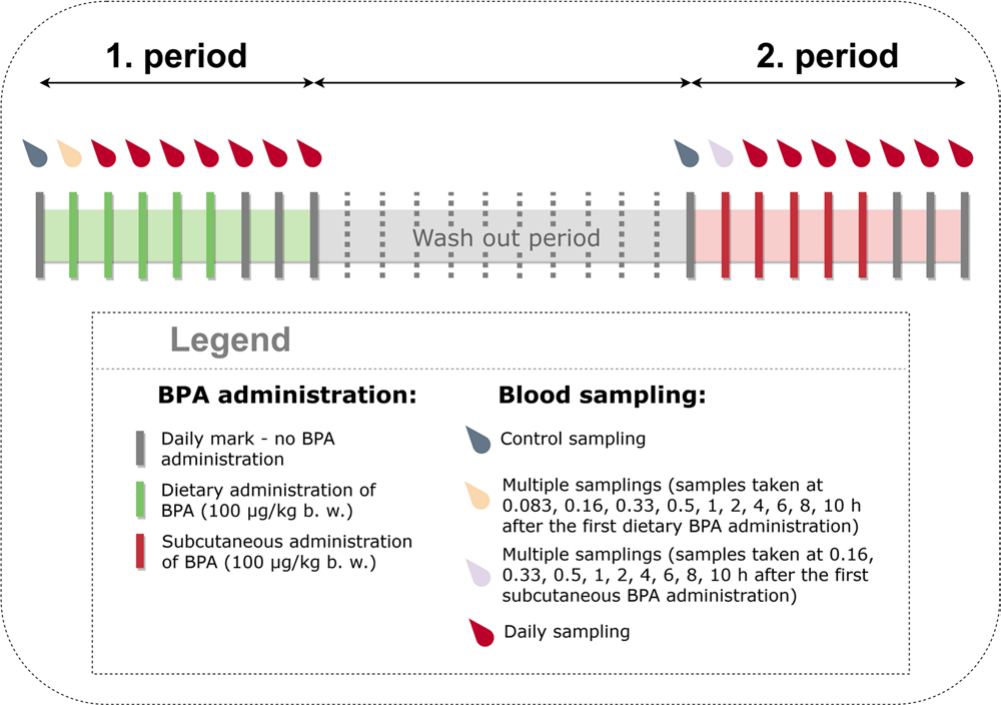
Study design with two experimental periods, BPA administration and blood sampling schedule for the ewe.

A diagram illustrating the design of the study, including the two experimental periods, BPA administration and blood sampling schedule of the ewe, is provided below (Fig. 1).

Milk sampling was done in both periods. The ewe was hand-milked. On the first day of the experiment milk was collected six and 10 hours after the first BPA administration and every next day just before the following administration. Before the first administration in each period the ewe was milked and then the lamb was separated from the ewe during next six hours to allow estimation of the amount of BPA excreted in milk. The sampling period continued from the 5^th^ until the 8^th^ day of both periods, when there was no BPA administration. Milk was collected in PP containers and stored at −20 °C until analysis.

Blind samples of blood plasma from the sheep and suckling lamb and milk from the sheep were taken just before the start of both periods, to provide a baseline for the analysis.

The precautions taken to avoid contamination with BPA during sampling were: using glass vacuum tubes for blood collection and PP containers for milk collection.

### Free BPA and total BPA sample analysis

BPA stock solution of 200 µg/mL was prepared in acetonitrile, while the intermediate and working standard solutions ranging from 2,000 to 1.0 ng/mL were further prepared in a mixture of acetonitrile and water at a ratio of 35: 65 (v/v). Working standard solutions ranging from 50,000 to 50 ng/mL for fortification of the total BPA samples were prepared in water with a small portion (≤20%, v/v) of ethanol or acetonitrile. All solutions were prepared using high purity deionised water obtained using a PureLab Option and PureLab Classic water purification system (Elga, Woodridge, Illinois, USA). The acetonitrile and methanol used were of HPLC gradient grade purity and purchased from J.T. Baker (Center Valley, PA, USA). Only high quality glass or labware were used for the sample analysis to avoid contamination with BPA during analysis.

Samples of the sheep blood plasma and milk were tested for the presence of both free (unconjugated) and total BPA (a sum of free and conjugated, mostly in a form of BPA-GLUC), of which the latter was determined indirectly by enzymatic conversion of the BPA-GLUC to free BPA. Sample aliquots of 1.5 and 5 mL were taken for the analysis of free BPA in the blood plasma and milk, respectively, while aliquots of 1.0 and 2.5 mL were taken to determine the total BPA and were diluted by 1.1 M Na-acetate buffer solution with pH values of 5.3 and 5.1 and volumes of 1.0 and 2.5 mL for the blood plasma and milk, respectively. Forty and 70 µL of ß-glucuronidase from *Helix pomatia*, type HP-2, ≥100,000 units/mL including also ≤7,500 sulfatase units/mL (Merck, Sigma-Aldrich, Darmstadt, Germany) were added to each sample of the blood plasma and milk, respectively. Samples were then incubated in a shaking water bath at 37 °C for 4 h.

The blood plasma and milk samples were further extracted by 6 and 10 mL of acetonitrile, respectively and ultrasonicated before being evaporated to dryness at 40–42 °C under a stream of N_2_ using an N-evap 111 evaporator (Organomation Associates, Berlin, MA, USA). A further clean-up procedure included solid phase extraction (SPE) by the use of molecularly imprinted polymer (MIP) columns AFFINIMIP SPE Bisphenols, 6 mL, 100 mg (AFFINISEP, Petit-Couronne, France), while the additional use of a Chromabond HR-X phase, with 6 mL columns, 200 mg, and 85 µm particle size (Macherey-Nagel, Düren, Germany) was previously utilised for all deconjugated sample extracts, as described by Deceuninck *et al*.^17^. Final SPE extracts were re-dissolved in acetonitrile/H_2_O (35/65, v/v) as follows: both free BPA blood plasma and milk samples in 0.5 mL, and total BPA blood plasma and milk samples in 1.0 and 0.5 mL, respectively. Fifty µL of the final extract were taken for the HPLC analysis.

HPLC measurements were performed using a Varian ProStar HPLC system (Varian Analytical Instruments, Walnut Creek, CA, USA), comprised of a tertiary pump (240 model), automatic injector (410 model), fluorescence detector (363 model), degasser and Galaxie 1.7.4.5 analytical software. Chromatographic separation was performed at room temperature by the gradient binary pumping of water and acetonitrile at a flow rate of 1 mL/min through a Hypersil Gold C18 analytical column, 150 ×4.6 mm, with a particle size of 3 µm, which was protected with Hypersil GOLD 3 µ Drop in the guards (Thermo Scientific, Waltham, MA, USA). The mobile phase gradient was as follows: 0–2 min, 35% (v/v) of acetonitrile, gradient to 12 min, 35–50% (v/v) of acetonitrile, held to 20 min, gradient to 20.5 min, 50–35% (v/v) of acetonitrile, held to 21 min. The excitation and emission wavelengths of the fluorescence spectrophotometry analysis were set at 230 and 315 nm, respectively^18^. The results were evaluated in accordance with the external standard method using a standard calibration curve as a function of chromatographic peak areas and standard concentrations. Each sample series consisted of a matrix sample, obtained before the first periodic BPA administration (a baseline sample), five to seven animal study samples in duplicate and two baseline matrix samples fortified with BPA to control the recovery rate. The measured sample concentrations were corrected for the possible baseline matrix response and for the mean recovery of the respective series and then used as final results.

Validation of the analytical methodology used was performed to demonstrate its fitness for the stated purpose. Linearity was determined by the least-squares method to calculate regression and correlation parameters for six to seven standard concentration points per calibration curve (range 1.0–100 ng/mL), and for both matrices as a correlation between measured and added concentrations (ranges 0.25–10 μg/L and 1.0–50 μg/L for free and total BPA in blood plasma, respectively, 0.5–15 μg/L for both free and total BPA in milk). Mean recovery was evaluated by analysis of four to six fortified blank materials at two concentration levels at separate time points (blood plasma: free BPA 2 and 10 μg/L, total BPA 25 and 50 μg/L; milk: free BPA 2 and 5 μg/L, total BPA 5 and 10 μg/L). The within-laboratory reproducibility of the method was evaluated as the coefficient of variation (CV) of the determined and recovery values. The limit of detection (LOD) value was estimated as the BPA concentration in the retention time window where the analyte was to be expected, which corresponded to 3 × noise and was corrected for the blank matrix response.

### Toxicokinetic analysis

Each entity (free BPA, BPA-GLUC, and total BPA) plasma concentration time course until the second BPA administration was first analysed using a noncompartmental approach to obtain the estimates of the area under the concentration–time curve extrapolated to infinity (AUC), maximum concentration in plasma and time when it occurs (c_max_ and t_max_, respectively). AUC was calculated using the linear trapezoidal method and extrapolated to infinity by addition of the term C_last_/λ_z_, where C_last_ is the last quantified concentration measurement and λ_z_ is the terminal slope of the concentration profile in the semi-log plot calculated by linear regression. t_max_ and c_max_ were reported as observed. AUC values were used to estimate clearance (CL) as CL = Dose/AUC_sc_ and relative bioavailability after dietary administration (F_r_) as F_r_ = AUC_po_/AUC_sc_. The indexes po and sc refer to the route of administration (dietary and subcutaneous, respectively) and Dose is the single BPA dose (100 µg/kg b. w.). Note that CL can be estimated only after intravenous administration. Our estimate of CL is therefore apparent clearance, i.e. assuming complete bioavailability after subcutaneous administration.

Subsequently, all TK data after both routes of administration were simultaneously fitted to a one- and two-compartment model with first-order absorption and elimination. The estimated parameters were CL, volume of the central and peripheral compartment (Vc and Vp, respectively), distribution clearance (Q), absorption rate constants after subcutaneous and dietary administration (k_a sc_ and k_a po_, respectively) and relative bioavailability (F_r_). Parameter fitting was performed using ADAPT II software^19^ with the maximum likelihood method and a proportional variance model, V_i_ = (σ × Y_i_)^2^, where V_i_ is the variance of the i-th data point and Y_i_ is the value predicted by the model. The Akaike Information Criterion (AIC) value was used to select the model.

Permeation of free BPA, BPA-GLUC and total BPA into milk was modelled as a first order process dA_m_/dt = k_m_ × Cp(t), where dA_m_/dt is the transfer rate in µg/h, C_p_(t) is the BPA plasma concentration at time t, and k_m_ is the transfer rate constant. k_m_ was estimated by simultaneous fitting of the amounts excreted into milk up to six hours after the first subcutaneous and dietary administration, with TK parameters for the plasma data fixed to previously estimated values. The amounts excreted in milk up to 6 h were approximated by multiplication of the concentration in milk at 6 h by 0.25 L, i.e. assuming an average milk yield of 1 L/day. We tested the hypothesis that only free BPA is transferred into milk and subsequently conjugated in the mammary gland, i.e. fixing the TK parameters to the values estimated for the free BPA versus the hypothesis that BPA-GLUC is also transferred, i.e. fixing the TK parameters to the values obtained for the conjugated and total BPA.

## Results

### Validation of the analytical methodology used

The validation parameters of the BPA blood plasma and milk analysis are presented in Table 1. The method was linear for BPA standards and matrices, as proved by the determination coefficients (r^2^) of ≥0.999 and ≥0.991, respectively. Mean recoveries for free and total BPA in the blood plasma were 82.3 and 49.5%, respectively and in the milk 62.9 and 54.3%, respectively. The total BPA refers to the sum of free and BPA-GLUC. The CVs of the concentrations detected and recovery in the fortified samples were from 1.5–24.4% under within-laboratory reproducibility conditions. The LOD values were 0.05–0.1 µg/L and 0.2–0.4 µg/L for the free and total BPA determination, respectively, and differed according to a more comprehensive chromatographic background in the total BPA extracts.

**Table 1:** Validation results of BPA determination in blood plasma and milk.

Parameter		Free BPA				Total BPA				
		Blood plasma		Milk		Blood plasma			Milk	
**Linearity**										
Standards	Range (ng/mL)	1.0‒100								
	Correlation (r^2^)	0.9993‒0.9999								
Matrix	Range (µg/L)	0.25‒10		0.5‒15		1.0‒50			0.5‒-15	
	Correlation (r^2^)	0.9956		0.9984		0.9982			0.9908	
**Recovery and precision**	Added concentration (µg/L)	2	10	2	5	25		50	5	10
	Recovery (s.d.) (%)	76.13 (2.26)	88.37 (1.29)	56.76 (13.83)	69.08 (11.90)	57.11 (13.64)		41.96 (2.99)	57.67 (8.53)	51.04 (10.61)
	CV (%)	2.97	1.46	24.36	17.23	23.88		7.13	14.79	20.78
**LOD (µg/L)**		0.05		0.1		0.4			0.2	

### TK analysis

BPA levels were checked before conducting both the first and second part of the experiment to provide a baseline for the analysis. The ewe entered the first part of the experiment with 0.05 and <0.4 µg/L of the free and total BPA in the blood plasma, respectively, and with <0.1 and 0.31 µg/L of the free and total BPA in milk, respectively. Just before the start of the subcutaneous administration, the ewe’s blood plasma contained 0.15 and 0.72 µg/L of the free and total BPA, respectively, while its milk contained <0.1 and 0.35 µg/L of the free and total BPA, respectively.

### Comparison of the plasma concentration-time profiles

The maximum plasma concentration of free BPA obtained after subcutaneous administration was higher than after dietary administration. In addition, free BPA exposure was prolonged after subcutaneous administration compared to the dietary route of intake. With the dietary route, c_max_ of free BPA in plasma was 2.15 µg/L and was obtained very quickly, at 0.33 h. For the subcutaneous route, c_max_ of free BPA was 6.41 µg/L and was obtained after 2 h.

The c_max_ values of BPA-GLUC were similar for both routes of exposure and were 49.64 µg/L (t_max_ = 1 h) for subcutaneous administration and 41.3 µg/L (t_max_ = 0.33 h) for dietary administration.

The same seems to be valid for the c_max_ of total BPA, where c_max_ after subcutaneous administration was 55.6 µg/L (t_max_ = 1 h) and c_max_ after dietary administration was 43.46 µg/L (t_max_ = 0.33 h).

The AUC of free BPA was 33.3 µg h/L for subcutaneous administration and 1.28 µg h/L for dietary administration. However, for BPA-GLUC and total BPA the AUC was 274 µg h/L and 307 µg h/L for subcutaneous administration and 409 µg h/L and 410 µg h/L for dietary administration, respectively.

Administration by the subcutaneous route led to a higher overall internal exposure to free BPA and lower internal exposure to BPA-GLUC/total BPA compared to the dietary route.

Clearance and relative bioavailability of free BPA, BPA-GLUC and total BPA obtained with noncompartmental TK analysis are presented in Table 2.

**Table 2:** CL = clearance, F_r_ = relative bioavailability, dietary vs. subcutaneous administration. TK parameters of the noncompartmental TK analysis following the first dietary and subcutaneous BPA administration.

Parameter		Free BPA	BPA-conjugate	Total BPA
Subcutaneous administration	CL (L/h/kg)	3.0055	0.3650	0.3258
Dietary administration	F_r_ (%)	3.8	149	134

The TK parameters obtained with the TK model of the free BPA, BPA-GLUC and total BPA are gathered in Table 3. Blood plasma BPA-GLUC and total BPA concentration time courses were described with a one-compartment model, while a two-compartment model was more suitable for free BPA.

**Table 3:** CL = clearance, V_c_ = volume of central compartment, k_a sc_ = absorption rate constant after subcutaneous administration, Q = distribution clearance, V_p_ = volume of peripheral compartment, k_a po_ = absorption rate constant after dietary administration, F_r_ = relative bioavailability, dietary vs. subcutaneous administration, RSE = relative standard error, calculated by dividing standard error by mean value (%). TK parameters of the BPA (RSE%) following repeated dietary and subcutaneous administration.

Parameter	Free BPA	BPA-GLUC	Total BPA
CL (L/h/kg)	3.12 (7.2%)	0.388 (9.9%)	0.343 (9.1%)
V_c_ (L/kg)	2.45 (23.4%)	2.17 (13.3%)	1.89 (12.4%)
k_a sc_ (h^−1^)	0.455 (16.1%)	2.57 (29.1%)	2.59 (27.1%)
Q (L/h/kg)	0.425 (39.4%)	/	/
V_p_ (L/kg)	5.75 (27.4%)	/	/
k_a po_ (h^−1^)	6.39 (67.1%)	3.24 (19.2%)	3.44 (20.0%)
Fr (%)	4.52 (29.9%)	115 (12.9%)	101 (12.4%)

**Figure 2:**
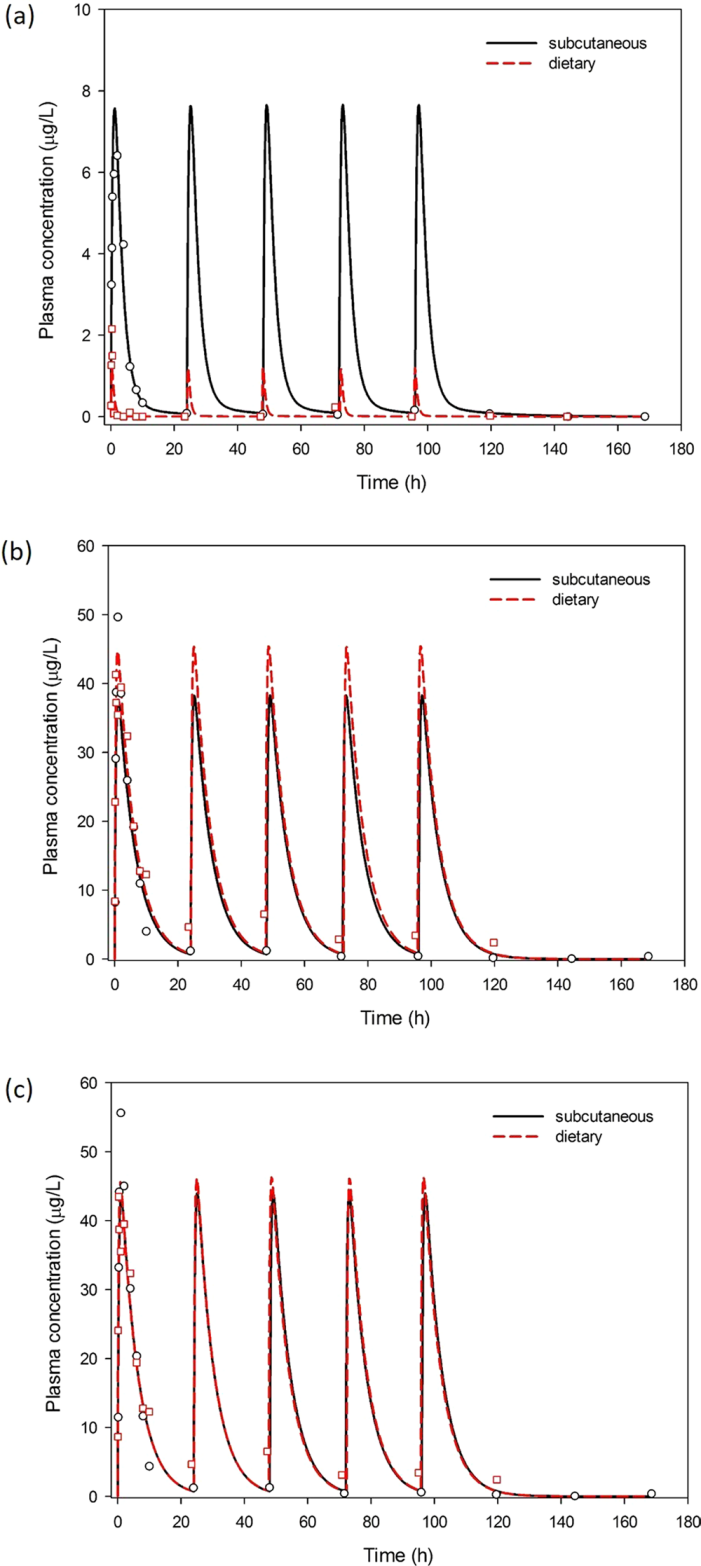
Time course of free BPA (**a**), BPA-GLUC (**b**) and total BPA (**c**) plasma concentration.

Figure 2 shows the plasma free BPA, BPA-GLUC and total BPA concentration-time profiles after repeated dietary and subcutaneous exposure of the ewe during the five days of a daily BPA administration of 100 µg/kg b. w. and the subsequent three days of no BPA administration. The concentration-time profiles between the two routes varied slightly for BPA-GLUC and total BPA, and markedly for the free BPA.

The ewe was administered 100 μg/kg b. w./day of BPA sc or po for five days, one time per day in the morning. The administration was stopped the sixth day of the experiment, while the sampling continued until the eighth day. Blood samples were collected at 0.08 – 0.17 – 0.33 – 0.5 – 1 – 2 – 4 – 6 – 8 – 10 – 23.33 – 46.58 – 69.35 – 92.35 – 116.1 – 140.1 – 164.1 hours after the first dietary administration and 0.17 – 0.33 – 0.5 – 1 – 2 – 4 – 6 – 8 – 10 – 23.92 – 47.84 – 71.34 – 95.17 – 118.67 – 143 – 167.5 hours after the first subcutaneous administration.

### Estimation of the free BPA, BPA-GLUC and total BPA elimination into the milk

Given our model assuming passive transfer (first-order) of BPA into milk, it is more likely that only free BPA is transferred into the mammary gland (AIC = −5.066 for the BPA-GLUC and AIC = −5.031 for the total BPA), versus the hypothesis that BPA-GLUC is also transported (AIC = −3.900 for the BPA-GLUC and AIC = −2.254 for the total BPA). The estimated rate constants of transfer into milk (estimate (RSE%)) were 0.00832 L/h (0.1%) for the free BPA, 0.01839 L/h (3.6%) for the BPA-GLUC and 0.02669 L/h (2.4%) for the total BPA.

For the first part of the experiment (dietary administration), the percentage of the dose eliminated with milk was 0.0002% (RSE = 0.1%) for the free BPA, 0.00045% (RSE = 3.6%) for BPA-GLUC and 0.00066% (RSE = 24%) for total BPA.

For the second part of the experiment (subcutaneous administration), the percentage of the dose eliminated with milk was 0.00453% (RSE = 0.1%) for the free BPA, 0.01001% (RSE = 3.6%) for BPA-GLUC and 0.01452% (RSE = 2.4%) for total BPA.

Regarding the suckling lamb, which drank milk after its mother was administered with BPA by the dietary or subcutaneous routes, there were only traces of BPA in the samples of its plasma.

## Discussion

The purpose of this study was to investigate the TK of BPA and to evaluate its elimination into the sheep milk after two different routes (po and sc) of repeated low dose BPA administration.

A comparison of the plasma concentration-time profile for the basic TK parameters of the two administration routes was made using the noncompartmental approach. Regarding the comparison of both routes of BPA administration, our preliminary findings are similar to those of Guignard *et al*.^20^, where the TK parameters for the same routes of administrations but with higher dose regimens were compared. The formulations for the dietary and as well for subcutaneous route of administration were similar in both studies. In our study, the c_max_ of free BPA for dietary administration was obtained quickly (0.33 h). In their study, mean c_max_ was attained 0.12 h for two ewes and 0.20 h for two others. For the subcutaneous route, c_max_ in our study was obtained after 2 h, in their study it was obtained after 2 h for three ewes and after 1 h for one ewe. In our study, the free BPA c_max_ for the subcutaneous route was three-fold higher than for the dietary route and in their work the free BPA c_max_ for the subcutaneous route was 4.6 ± 1.5-fold higher than for the dietary route. Our study demonstrates a higher cumulative (AUC) internal exposure to free BPA after subcutaneous administration compared to the dietary route, which is in line with the findings of Guignard *et al*.^20^. In their study, the relative bioavailability of BPA for the dietary as compared to subcutaneous route was 3.3 ± 0.3%. In our work, the relative bioavailability of BPA for the dietary as compared to subcutaneous route was 4.5%. Both this earlier work and the current study were also in agreement with regard to the BPA-GLUC concentration time course. Unlike free BPA, the BPA-GLUC concentration time courses are very similar for the two routes of exposure. Although our preliminary study was made with only one animal, the acquired data are in agreement with those of Guignard *et al*.^20^. This is important, as measured concentrations from our study were the base for the TK model, which we used to evaluate the elimination of BPA into the sheep milk. Sampling of the milk was possible only at a couple of sampling points, and thus it was not possible to make time-concentration profiles for it. However, the measurments of BPA and BPA-GLUC in blood plasma and our TK model enabled us to estimate the percentage of the dose eliminated with milk, which was less than 0.1% for free BPA, BPA-GLUC and total BPA, regardless of the route of administration. This result is comparable with the results of Snyder *et al*., where they found only a small fraction of the ^14^C labelled BPA (0.63 +/− 0.13 µg/equiv/mL) 8 hours after dosing^10^. Regarding free BPA, BPA-GLUC and total BPA, it is already indirectly proven in rats that free BPA is transferred into the mammary gland to a greater extent than BPA-GLUC^13,21^. Given our TK models, the same was true in our study for the ewe. In the first model we were assuming passive transfer (first-order) of free BPA into milk, and in the second we were assuming that BPA-GLUC would also be transported. Based on the lower value of the Akaike information criterion (AIC), which is one of the indexes, which are showing model’s goddness of fit, in the first model, it is more likely that only free BPA is transferred into the mammary gland. Nevertheless, it was reported that the major molecular species in the milk of rats after oral administration of ^14^C-BPA was BPA-GLUC. The concentrations measured in milk six hours after BPA administration (dietary and subcutaneous) in our study show the same result. Six hours after dietary administration, the concentration of free BPA was 0.05 µg/L and the concentration of BPA-GLUC was 0.78 µg/L. Similarly, the concentrations of free BPA and BPA-GLUC after subcutaneous administration were 0.87 and 1.89 µg/L, respectively. Regarding the BPA-GLUC in the milk, we hypothesise that free BPA is passively transferred into the mammary gland, and subsequently conjugated in its glucuronidated form presumably by the uridine 5′-diphospho-glucuronosyltransferase (UDP-glucuronosyltransferases) in the mammary gland. Only a few studies have evaluated UDP-glucuronosyltransferases (UGTs) presence in breast tissue. Expression of UGT2B10, UGT2B11, UGT2B15 and UGT2B UGT1A10 and UGT2B7, and UGT2B11 enzymes have been proven in humans, and the results of Street *et al*. confirm the capability of glucuronidation of BPA in human breast tissue, although with glucuronidation activities that are much lower (by more than 100,000-fold) compared with those seen in the liver^22^. There are currently no (to the best of our knowledge) known studies that have evaluated the presence of UDP-glucuronosyltransferases in the ewe mammary gland, although it seems reasonable to assume that the mammary glands of all mammals are equipped with comparable detoxifying mechanisms.

The above mentioned concentrations of free BPA measured in this study are well within the range with the concentrations found in raw milk measured in a recent Italian monitoring study, where the concentrations of free BPA ranged from 0.081–2.492 µg/L^23^. However, the concentrations measured in commercial milk samples were generally higher (from 14.0 to 521.0 µg/L)^6^, meaning that the BPA load in consumption milk is greater at the end of the production line and during further processing.

We report that three years after the end of the experiment, the ewe and the lamb are in a good health condition. There were no reproductive abnormalities reported in the ewe in the past three years. After the experiment, the ewe had offspring each year. In the year 2018, the offspring were two healthy male lambs, while in the year 2019, the offspring were one healthy male and one healthy female lamb. The grown-up lamb developed normally, has been recently mated for the first time and is currently pregnant (12. 3. 2020). Both animals have appropriate body weight and there is no known history of disease.

## Conclusion

In this preliminary study, we are presenting the detected BPA and BPA-GLUC concentrations in the milk samples obtained from the ewe administered by two different routes of administration. With the TK model, on the basis of blood plasma and milk measurements, we were able to estimate the percentage of the eliminated BPA in the milk, which is minimal regardless of the administered route of BPA. In addition, with our TK model we can suggest that in sheep, being a large animal model, BPA is transferred in the milk in the mammary gland mostly in its free form. As BPA-GLUC concentrations were higher than free BPA concentrations in milk, and that with regards to our TK model, only BPA can cross the barrier between blood and mammary gland, we are estimating that free BPA is probably subsequently metabolised in mammary gland. Further studies are needed to confirm preliminary findings in this study, especially in a view of greater number of animals, different phases of lactation and different BPA doses.

## Supplementary information

Supplementary information.
