# Supplementary material for: Preliminary toxicokinetic study of BPA in lactating dairy sheep after repeated dietary and subcutaneous administration
Source: Sci Rep. 2020 Apr 16;10:6498. doi: 10.1038/s41598-020-63286-z (PMC7162867; doi:10.1038/s41598-020-63286-z)
Supplement: Supplementary file 1 — Supplementary information. [file 41598_2020_63286_MOESM1_ESM.docx]

Supplementary information

**Preliminary toxicokinetic study of BPA in lactating dairy sheep after repeated dietary and subcutaneous administration**

**Sabina Šturm^1,*^, Iztok Grabnar^2^, Andrej Škibin^3^, Milan Pogačnik^1^, Vesna Cerkvenik-Flajs^1^**

Supplementary Table S1

| Clinical examination of the sheep | | | | |
| --- | --- | --- | --- | --- |
| Date | Body temperature | Pulse rate (per minute) | Breathing frequency  (per minute) | Rumination frequency (per five minutes) |
| 20.3.2017 (1^st^ day of the first period of the experiment) | 38.7 °C | 65 | 16 | 10 |
| 7.4.2017 (1^st^ day of the second period of the experiment) | 38.8 °C | 72 | 20 | 10 |

Supplementary Table S2

| Haematology | | |  |  |
| --- | --- | --- | --- | --- |
|  | Parameter | Value | Date of blood sampling | Date of performed analysis |
|  | Erythrocytes (x10^12^/L) | 8.80 | 20.3.2017 | 21.3.2017 |
|  | Hb (g/dL) | 9.6 |  |  |
|  | MCV (fL) | 37 |  |  |
|  | Ht (%) | 32.2 |  |  |
|  | Leukocytes (x10^9^/L) | 8.1 |  |  |
|  | MCH  (pg) | 10.9 |  |  |
|  | MCHC  (g/dL) | 29.7 |  |  |
|  | Platelets (x10^9^/L) | 509 |  |  |
|  | Neutrophils (%) | 31 |  |  |
|  | Eosinophils (%) | 1 |  |  |
|  | Basophils (%) | 0 |  |  |
|  | Lymphocytes (%) | 68 |  |  |
|  | Segmented neutrophils (%) | 0 |  |  |
|  | Monocytes (%) | 0 |  |  |

Haematological analyses were performed utilising the Scil Vet abc Plus (Horiba, Japan) automated haematological analyser. Differential white blood cell count was determined according to the standard procedure; smears were stained with Hemacolor (Merck, Darmstadt, Germany) and manually counted via microscopic examination.

Supplementary Table S3

| Biochemistry | | | | |
| --- | --- | --- | --- | --- |
|  | Parameter | Value | Date of blood sampling | Date of performed analysis |
|  | AST (U/L) | 171 | 20.3.2017 | 21.3.2017 |
|  | GGT (U/L) | 42 |  |  |
|  | iP (mmol/L) | 2.16 |  |  |
|  | Ca (mmol/L) | 2.54 |  |  |
|  | Mg (mmol/L) | 1.02 |  |  |
|  | Na (mmol/L) | 152 |  |  |
|  | K (mmol/L) | 4.51 |  |  |
|  | Cl (mmol/L) | 109 |  |  |
|  | Gluc (mmol/L) | 3.0 |  |  |
|  | Urea (mmol/L) | 4.80 |  |  |
|  | Crea (µmol/L) | 62 |  |  |
|  | Fe (µmol/L) | 24.9 |  |  |

For biochemistry analysis, biochemistry analyser RX Daytona (Randox Laboratories Ltd, Crumlin; UK) was utilized.
